# Cisplatin and phenanthriplatin modulate long-noncoding RNA expression in A549 and IMR90 cells revealing regulation of microRNAs, Wnt/β-catenin and TGF-β signaling

**DOI:** 10.1038/s41598-021-89911-z

**Published:** 2021-05-17

**Authors:** Jerry D. Monroe, Satya A. Moolani, Elvin N. Irihamye, Katheryn E. Lett, Michael D. Hebert, Yann Gibert, Michael E. Smith

**Affiliations:** 1 Department of Cell and Molecular Biology, Cancer Center and Research Institute, University of Mississippi Medical Center, Jackson, MS 39216 USA; 2 Biology Department, Western Kentucky University, Bowling Green, KY 42101-1080 USA; 3 Present Address: Program in Cognitive Science, Case Western Reserve University, Cleveland, OH 44106-7063 USA; 4 Present Address: Program in Neuroscience, Indiana University Bloomington, Bloomington, IN 47405-2204 USA

**Keywords:** Cancer, Computational biology and bioinformatics, Drug discovery, Molecular biology

## Abstract

The monofunctional platinum(II) complex, phenanthriplatin, acts by blocking transcription, but its regulatory effects on long-noncoding RNAs (lncRNAs) have not been elucidated relative to traditional platinum-based chemotherapeutics, e.g., cisplatin. Here, we treated A549 non-small cell lung cancer and IMR90 lung fibroblast cells for 24 h with either cisplatin, phenanthriplatin or a solvent control, and then performed microarray analysis to identify regulated lncRNAs. RNA22 v2 microRNA software was subsequently used to identify microRNAs (miRNAs) that might be suppressed by the most regulated lncRNAs. We found that miR-25-5p, -30a-3p, -138-5p, -149-3p, -185-5p, -378j, -608, -650, -708-5p, -1253, -1254, -4458, and -4516, were predicted to target the cisplatin upregulated lncRNAs, IMMP2L-1, CBR3-1 and ATAD2B-5, and the phenanthriplatin downregulated lncRNAs, AGO2-1, COX7A1-2 and SLC26A3-1. Then, we used qRT-PCR to measure the expression of miR-25-5p, -378j, -4516 (A549) and miR-149-3p, -608, and -4458 (IMR90) to identify distinct signaling effects associated with cisplatin and phenanthriplatin. The signaling pathways associated with these miRNAs suggests that phenanthriplatin may modulate Wnt/β-catenin and TGF-β signaling through the MAPK/ERK and PTEN/AKT pathways differently than cisplatin. Further, as some of these miRNAs may be subject to dissimilar lncRNA targeting in A549 and IMR90 cells, the monofunctional complex may not cause toxicity in normal lung compared to cancer cells by acting through distinct lncRNA and miRNA networks.

## Introduction

**Figure 1:**
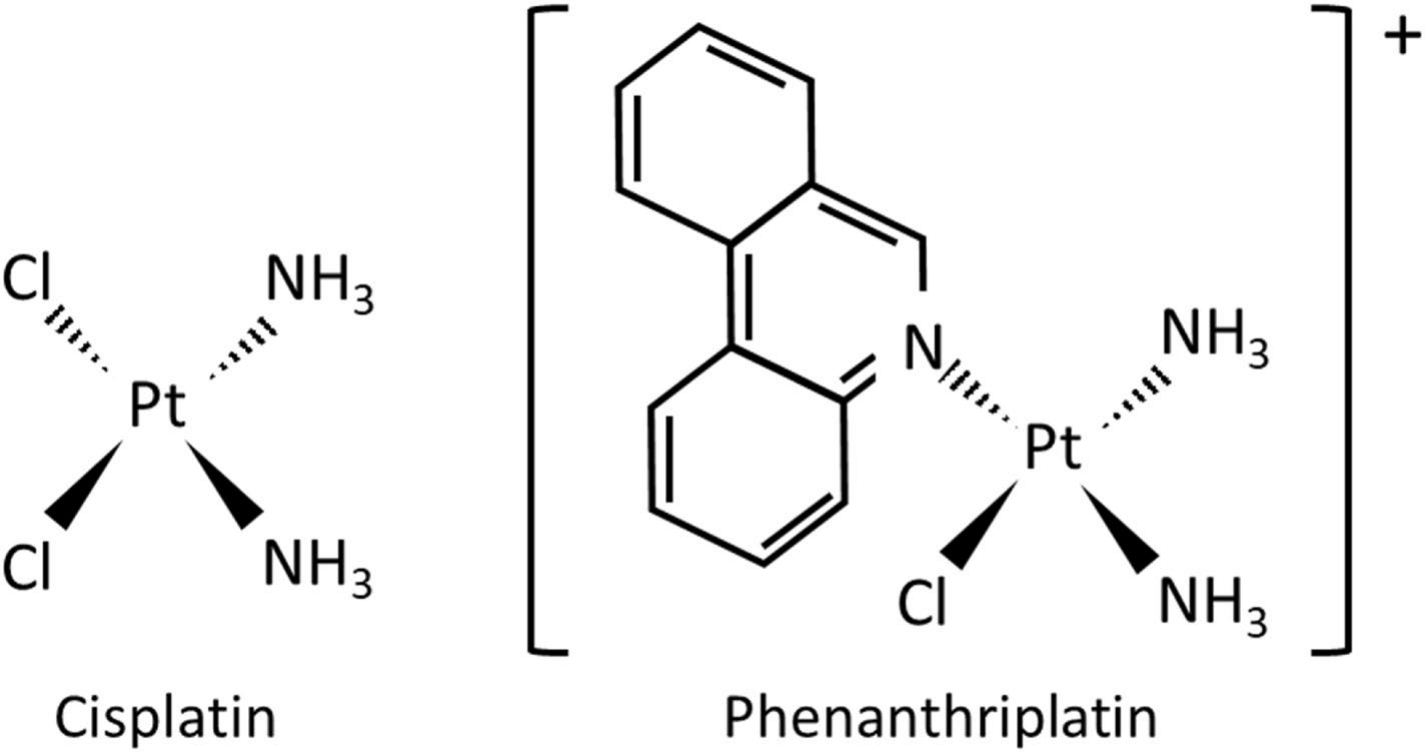
Chemical structures of cisplatin and phenanthriplatin. Cisplatin is a bifunctional platinum(II) complex with two chloride leaving ligands. The monofunctional platinum(II) complex, phenanthriplatin, has one chloride leaving ligand, and steric bulk in the form of a heterocyclic phenanthridine ligand placed *cis* to the platinum coordinating site.

The platinum(II) complex, *cis*-diamminedichloroplatinum(II) (cisplatin; Fig. 1), is a bifunctional chemotherapy drug that upon aquation of its two chloride leaving ligands and entry into cancer cell nuclei, typically forms intrastrand crosslinks with DNA causing the nucleotide strands to bend and alteration of gene transcription^1,2^. As cisplatin chemotherapy is frequently associated with severe side-effects^3–6^, there has been considerable interest in identifying alternative platinum complexes that are toxic to cancer cells without causing severe side-effects. The monofunctional platinum(II) complex, cis-[Pt(NH_3_)_2_Cl (phenanthridine)]^+^ (phenanthriplatin; Fig. 1) has similar or superior anticancer efficacy as cisplatin in lung, breast, prostate, testicular, colorectal, glioblastoma and cervical cell lines^7,8^. Unlike cisplatin, monofunctional compounds have only one chloride leaving ligand and bind to a single strand of DNA without causing DNA to bend^7,9^. Studies have shown that phenanthriplatin can prevent RNA transcription and has a distinct DNA residue binding profile compared to cisplatin^7,10^.

Microarray studies suggest that cisplatin can regulate genes associated with drug resistance, metabolism, cell proliferation, apoptosis, cell adhesion, stress response, cell cycle control and DNA repair^11–14^. Long non-coding RNAs (lncRNAs) are RNAs more than 200 nucleotides in length and may interact with protein, chromatin and RNA targets where they can modulate epigenetic, transcriptional, and post-transcriptional function^15,16^. LncRNA expression can become dysregulated in lung cancer tissue, where their aberrant expression can promote tumor cell growth, apoptosis, invasion, and metastasis^15^. MicroRNAs (miRNAs) are non-coding RNAs that function post-transcriptionally by binding to messenger RNA (mRNA) to prevent transcription or promote mechanisms that increase mRNA degradation and can regulate tumorigenesis and metastasis^16^. Further, microarray analysis of lung cancer cells has shown that cisplatin can alter the expression of a lncRNA which acts as a regulatory sponge for a miRNA and modulates chemosensitivity^17^. Other studies conducted with microarrays in cisplatin-treated cells have shown that lncRNAs are involved in the regulation of cisplatin sensitivity in non-small cell lung cancer (NSCLC)^18–20^. However, the effect of monofunctional platinum(II) complexes on lncRNA expression and their potential regulatory interaction with miRNAs has not yet been characterized.

This study used microarray analysis to investigate whether phenanthriplatin utilizes different lncRNA signaling than cisplatin. The A549 non-small cell lung cancer (NSCLC) cell line is routinely used to evaluate platinum-based gene modulation, and the non-cancer lung fibroblast cell line, IMR90, has been used to assess cisplatin’s effect on gene regulation^21,22^. Here, we treated A549 and IMR90 cells with the monofunctional complex, cisplatin or a negative media only control. Then, RNA samples were extracted from the cell lines and subjected to microarray analysis. Next, lncRNAs found in the microarray results were bioinformatically analyzed to identify miRNAs with high targeting homology with the lncRNA sequences. qRT-PCR analysis was also used to validate the microarray results and identify miRNAs subject to distinct modulation by the platinum compounds. We found that phenanthriplatin treatment caused up- and downregulation of lncRNAs that were not modulated by cisplatin in either A549 or IMR90 cells. Also, in A549 cells, lncRNAs downregulated by the monofunctional complex have several potential miRNA binding partners associated with Wnt/β-catenin and transforming growth factor-β (TGF-β) signaling that were predicted to also target lncRNAs that were upregulated by cisplatin treatment. This result suggests that cisplatin and phenanthriplatin may utilize distinct lncRNA and miRNA networks to promote anticancer function. Further, we found that phenanthriplatin modulated different lncRNAs in IMR90 cells and that some of the miRNAs predicted to cause cell toxicity during phenanthriplatin treatment in A549 NSCLC cells might promote an opposite effect in IMR90 fibroblast cells.

## Results

### Microarray and lncRNA qRT-PCR results

Affymetrix microarray analysis was performed on samples taken from cisplatin and phenanthriplatin treated A549 and IMR90 cells to identify regulated lncRNAs. We used a fold change threshold of ≥ 1.5 to identify the most up- and downregulated lncRNAs in A549 cells treated with cisplatin or phenanthriplatin compared to control (Table 1).

**Table 1:** Top up- and downregulated lncRNAs in cisplatin or phenanthriplatin treated A549 cells versus control (N = 4; *p* ≤ 0.05; fold change ≥ 1.5).

↑lncRNA	*p*-value	Fold change	↓lncRNA	*p*-value	Fold change
**A549 cisplatin versus control treatment**					
IMMP2L-1	0.00891315	1.8955	RIPPLY3-5	0.00705943	− 2.19871
CBR3-1	0.00464401	1.83878	PTBP1-2	0.0067485	− 1.71431
ATAD2B-5	0.0193789	1.72105	PGPEP1L-3	0.00339978	− 1.70191
RPH3AL-1	0.000469075	1.6639	NRIP1-2	0.00183867	− 1.62827
MKLN1-18	0.016523	1.63835	NXPE2-7	0.00410451	− 1.62084
SPOPL-1	0.0202125	1.51837	ATIC-8	0.0295747	− 1.51791
INHBA-1	0.0160087	1.5156	CNPY1-10	0.00973523	− 1.49064
ANLN-1	0.00917173	1.51019	ZPBP-4	0.0191988	− 1.45981
**A549 phenanthriplatin versus control treatment**					
TRIM55-1	4.16E − 09	2.39127	AGO2-1	8.31E − 10	− 4.3059
MRPL39-10	0.000153385	2.15346	SLC26A3-1	3.29E − 12	− 3.88619
RARB-1	0.011061	1.81916	PTBP1-2	6.36E − 06	− 2.7492
ZNF609-3	0.000727879	1.81342	COX7A1-2	1.61E − 11	− 2.30012
CHODL-4	0.00341257	1.80458	S100A1-2	1.18E − 05	− 2.25392
IGFL3-1	0.000218019	1.79193	NRIP1-2	1.00E − 05	− 2.12976
PHF14-3	0.00284805	1.78111	RIPPLY3-5	0.0139288	− 2.0372
IFNAR2-1	0.000391497	1.76313	LRRTM4-1	1.48E − 11	− 1.99336
GRID2IP-1	0.000120206	1.75057	CHAF1B-2	0.000134259	− 1.99203
CYS1-3	1.64E − 05	1.71069	ANAPC1-4	1.72E − 05	− 1.98051

Microarray analysis was also used to identify regulated lncRNAs in IMR90 cells treated with cisplatin or phenanthriplatin compared to control samples (Table 2).

**Table 2:** Top up- and downregulated lncRNAs in cisplatin or phenanthriplatin treated IMR90 cells versus IMR90 controls (N = 4; *p* ≤ 0.05; fold change ≥ 1.5).

↑lncRNA	*p*-value	Fold Change	↓lncRNA	*p*-value	Fold Change
**IMR90 cisplatin versus control treatment**					
GNG11-3	0.000198901	2.27427	HYPM-1	0.000114876	− 2.43193
ATAD2B-5	0.00477697	1.95365	LINC02246	0.0159639	− 1.99427
MRPS5-1	8.10E − 05	1.87463	FAM230A-2	0.000269091	− 1.93542
BOD1L1-3	0.00176748	1.85706	NLRP12-1	0.000578748	− 1.9269
ROCK2-2	0.000436844	1.74978	SUMF1-5	0.00980215	− 1.61883
LINC01317	0.0007781	1.69699	MBLAC1-1	0.0049991	− 1.5777
HS6ST1-1	0.0141027	1.66025	MRPL39-6	0.00406971	− 1.52821
CLEC18B-7	0.0063992	1.64079	SPRED2-2	0.0231861	− 1.51758
FSTL3-1	0.0182367	1.62948	C7orf57-6	0.0319739	− 1.50444
**IMR90 phenanthriplatin versus control treatment**					
TRIM55-1	2.44E − 15	4.86918	AGO2-1	4.55E − 14	− 9.01189
FAM237B-2	4.10E − 07	3.26514	HYPM-1	9.77E − 06	− 2.89824
TMEM243-1	1.41E − 08	2.35553	ARCN1-1	1.59E − 06	− 2.51905
RHOB-1	0.000168369	2.33006	LINC02246	0.00217521	− 2.47497
STPG4-1	0.000526294	2.00582	EVX2-4	2.99E − 05	− 2.39257
TMPRSS2-2	0.000412829	2.00123	FAM230A-2	1.68E − 05	− 2.26691
VPS26C-2	0.000214323	1.92335	SCAF4-2	0.00655979	− 2.08203
BOD1L1-3	0.00131994	1.8943	ACVR1-1	0.00360306	− 2.00722
PLPP2-3	1.11E − 05	1.83916	SLC51A-1	0.000385162	− 1.93934
FAM222B-3	0.00455282	1.81357	C4A-AS1	0.00797777	− 1.89487

**Figure 2:**
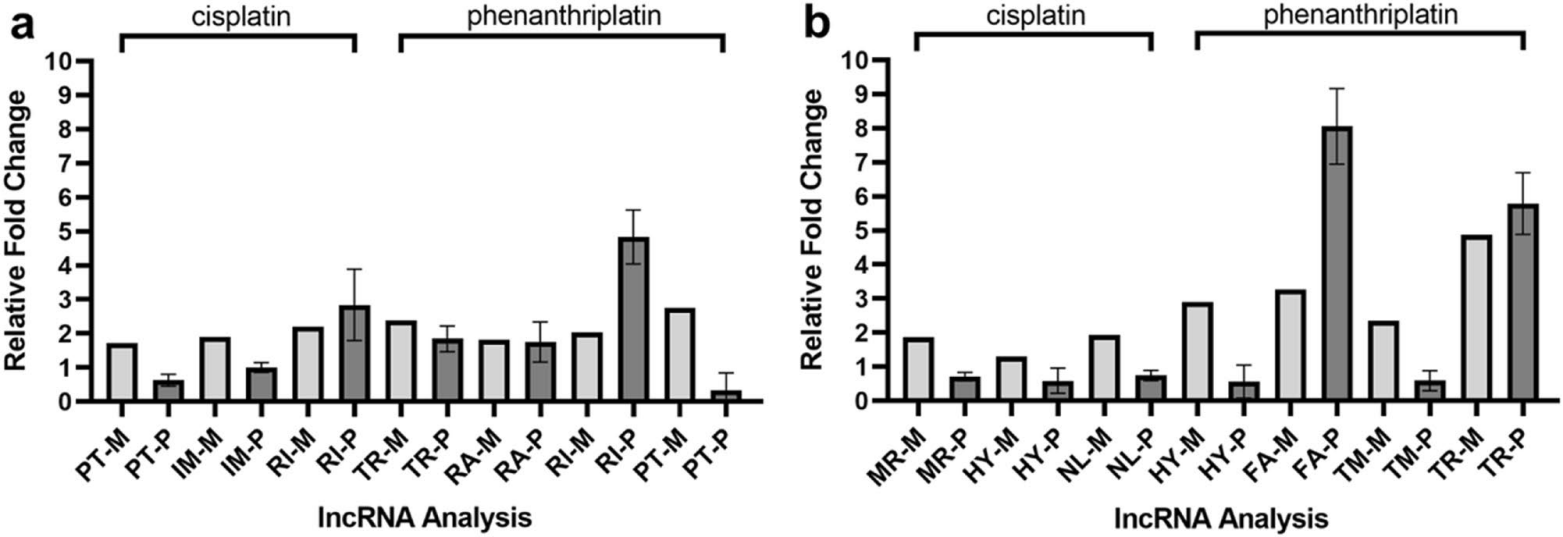
Validation of long non-coding RNA microarray expression data using qRT-PCR. (**a**) A549 cell line results for PTBP1-2 (PT), IMMP2L-1 (IM), RIPPLY3-5 (RI), TRIM55-1 (TR), and RARB-1 (RA). (**b**) IMR90 cell line results for MRPS5-1 (MR), HYPM-1 (HY), NLRP12-1 (NL), FAM237B-2 (FA), TMEM243-1 (TM), and TRIM55-1 (TR). Key: “-M” = microarray (light gray); “-P” = qRT-PCR (dark gray). Error bars represent mean ± standard deviation, N = 3.

To confirm the microarray data, we also performed qRT-PCR on samples taken from both the A549 and IMR90 cell lines and found that both methods detected similar expression profiles for many of the lncRNAs (Fig. 2). Cisplatin treated A549 samples had the following relative fold expression values (microarray compared to qRT-PCR respectively): PTBP1-2, 1.71–0.63; IMMP2L-1, 1.90–0.99; RIPPLY3-5, 2.20–2.71, which were within approximately one fold change value of one another or less (Fig. 2a). Phenanthriplatin treated A549 samples had very similar relative fold change values for two lncRNAs, TRIM55-1 (2.39–1.82) and RARB-1 (1.82–1.75), while RIPPLY3-5 (2.04–4.83) and PTBP1-2 (2.75–0.33) were within three relative fold change values of each other (Fig. 2a). For cisplatin treated IMR90 samples, relative fold expression values (microarray compared to qRT-PCR respectively) were within approximately one fold change value of one another: MRPS5-1 (1.87–0.71), HYPM-1 (1.30–0.59), NLRP12-1 (1.93–0.75), while several phenanthriplatin treated samples, HYPM-1 (2.90–0.57), FAM237B-2 (3.27–8.06), TMEM243-1 (2.36–0.59) differed by more than a fold change value, with the exception of TRIM55-1 (4.87–5.79), where relative expression was within a fold change value (Fig. 2b).

### Bioinformatics and miRNA qRT-PCR results

RNA22 v2 microRNA software was then used to identify microRNAs predicted to target the three most upregulated lncRNAs in A549 cells after cisplatin treatment. We found that cisplatin upregulated the lncRNAs, ATAD2B-5, CBR3-1, and IMMP2L-1, which may be targeted by several miRNAs with sequence homologies to lncRNAs downregulated by phenanthriplatin, including: AGO2-1, COX7A1-2 and SLC26A3-1 (Table 3; Supplementary Table S1). Also, we found that cisplatin downregulated the lncRNA, lnc-PGPEP1L-3, which is predicted to sponge miR-25-5p, a miRNA with high sequence homology to lnc-MRPL39-10, a lncRNA upregulated by phenanthriplatin (Table 3; Supplementary Table S1). These miRNAs are associated with Wnt/β-catenin and TGF-β signaling, e.g., miR-25-5p, -30a-3p, -138-5p, -149-3p, -185-5p, -378j, -608, -650, -708-5p, -1253, -1254 and -4516 (Table 3)^23–35^.

**Table 3:** MiRNAs projected to target regulated lncRNAs in A549 cisplatin and phenanthriplatin treated cells and predicted pathway signaling (*p* ≤ 0.05).

miRNA	Cisplatin lncRNAs	*p*-value	Phenanthriplatin lncRNAs	*p*-value	Predicted Pathway
miR-25-5p	CBR3-1 (Up)	0.000579	COX7A1-2 (Down)	0.0487	Wnt/β-catenin; TGF-β
miR-25-5p	PGPEP1L-3 (Down)	0.000077	MRPL39-10 (Up)	0.019	Wnt/β-catenin; TGF-β
miR-30a-3p	IMMP2L-1 (Up)	0.00333	SLC26A3-1 (Down)	0.0465	TGF-β
miR-138-5p	CBR3-1 (Up)	0.000579	SLC26A3-1 (Down)	0.0465	TGF-β
miR-149-3p	ATAD2B-5 (Up)	0.00675	AGO2-1, SLC26A3-1 (Down)	0.000001, 0.0465	Wnt/β-catenin
miR-185-5p	ATAD2B-5 (Up)	0.00675	AGO2-1 (Down)	0.000001	Wnt/β-catenin
miR-378j	CBR3-1 (Up)	0.000579	SLC26A3-1 (Down)	0.0465	Wnt/β-catenin; TGF-β
miR-608	ATAD2B-5 (Up)	0.00675	AGO2-1, COX7A1-2, SLC26A3-1 (Down)	0.000001, 0.0487, 0.0465	Wnt/β-catenin; TGF-β
miR-650	CBR3-1, IMMP2L-1 (Up)	0.000579, 0.00333	COX7A1-2, SLC26A3-1 (Down)	0.0487, 0.0465	Wnt/β-catenin
miR-708-5p	CBR3-1 (Up)	0.000579	SLC26A3-1 (Down)	0.0465	Wnt/β-catenin
miR-1253	IMMP2L-1 (Up)	0.00333	SLC26A3-1 (Down)	0.0465	Wnt/β-catenin
miR-1254	IMMP2L-1 (Up)	0.00333	COX7A1-2 (Down)	0.0487	Wnt/β-catenin
miR-4516	ATAD2B-5 (Up)	0.00675	AGO2-1 (Down)	0.000001	TGF-β

As signaling integrating lncRNAs and miRNAs in IMR90 cells might modulate distinct targets than those in A549 cells, we also identified microRNA targets of the three most upregulated lncRNAs after cisplatin treatment. We found that cisplatin upregulated the lncRNAs, ATAD2B-5, GNG11-3, and MRPS5-1, which may be targeted by miRNAs with sequence homologies to lncRNAs downregulated by phenanthriplatin, including: AGO2-1, ARCN1-1, HYPM-1 (Table 4; Supplementary Table S2). Cisplatin and phenanthriplatin treatment also downregulated the lncRNA, HYPM-1, which may sponge miR-608, and the monofunctional complex upregulated the lncRNA, TMEM243-1, with high sequence homology to miR-608 (Table 4; Supplementary Table S2). As in the A549 results, these miRNAs can potentially act through Wnt/β-catenin and TGF-β signaling (Table 4)^23,26,27,29,32–34,36^.

**Table 4:** MiRNAs projected to target regulated lncRNAs in IMR90 cisplatin and phenanthriplatin treated cells and predicted pathway signaling (*p* ≤ 0.05, except GNG11-3, *p* ≤ 0.0581).

miRNA	Cisplatin lncRNAs	*p*-value	Phenanthriplatin lncRNAs	*p*-value	Predicted pathway
miR-149-3p	GNG11-3 (Up)	0.0581	AGO2-1, ARCN1-1, HYPM-1 (Down)	0.000001, 0.00103, 0.0103	Wnt/β-catenin
miR-185-5p	ATAD2B-5 (Up)	0.00675	AGO2-1, ARCN1-1, HYPM-1 (Down)	0.000001, 0.00103, 0.0362	Wnt/β-catenin
miR-608	ATAD2B-5, MRPS5-1 (Up)	0.00675, 0.0151	AGO2-1, ARCN1-1, HYPM-1 (Down)	0.000001, 0.00103, 0.00156	Wnt/β-catenin; TGF-β
miR-608	HYPM-1 (Down)	0.0162	TMEM243-1 (Up)	0.0184	Wnt/β-catenin; TGF-β
miR-650	GNG11-3 (Up)	0.0581	HYPM-1 (Down)	0.0362	Wnt/β-catenin
miR-708-5p	ATAD2B-5 (Up)	0.00675	HYPM-1 (Down)	0.0162	Wnt/β-catenin
miR-4458	ATAD2B-5 (Up)	0.00675	AGO2-1, HYPM-1 (Down)	0.000001, 0.0103	TGF-β
miR-4516	ATAD2B-5 (Up)	0.00675	AGO2-1, HYPM-1 (Down)	0.000001, 0.0103	TGF-β

**Figure 3:**
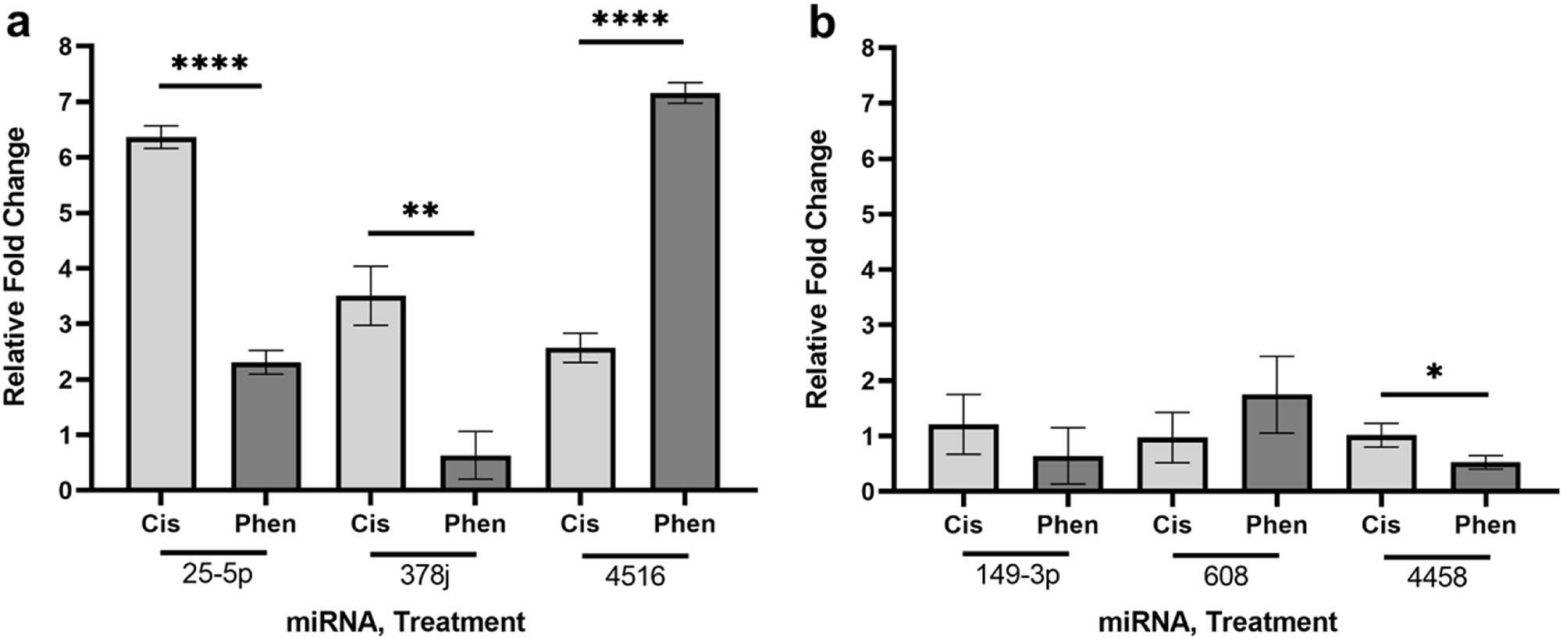
Measurement of miRNA expression using qRT-PCR. (**a**) A549 cell line results for miR-25-5p, -378j and -4516. (**b**) IMR90 cell line results for miR-149-3p, -608 and -4458. Key: “cis” = cisplatin; “phen” = phenanthriplatin. Error bars represent mean ± standard deviation, N = 3, “*” = *p* ≤ 0.05; “**” = *p* ≤ 0.01; “****” = *p* ≤ 0.0001.

To determine the expression of miRNA targets of lncRNAs identified in the bioinformatics analysis, we also performed qRT-PCR using locked nucleic acid primers on select miRNAs and found that cisplatin and phenanthriplatin treatment caused different regulation of three miRNAs, miR-25-5p, -378j, and -4516, in the A549 cell line and one miRNA, miR-4458, in the IMR90 cell line (Fig. 3).

**Figure 4:**
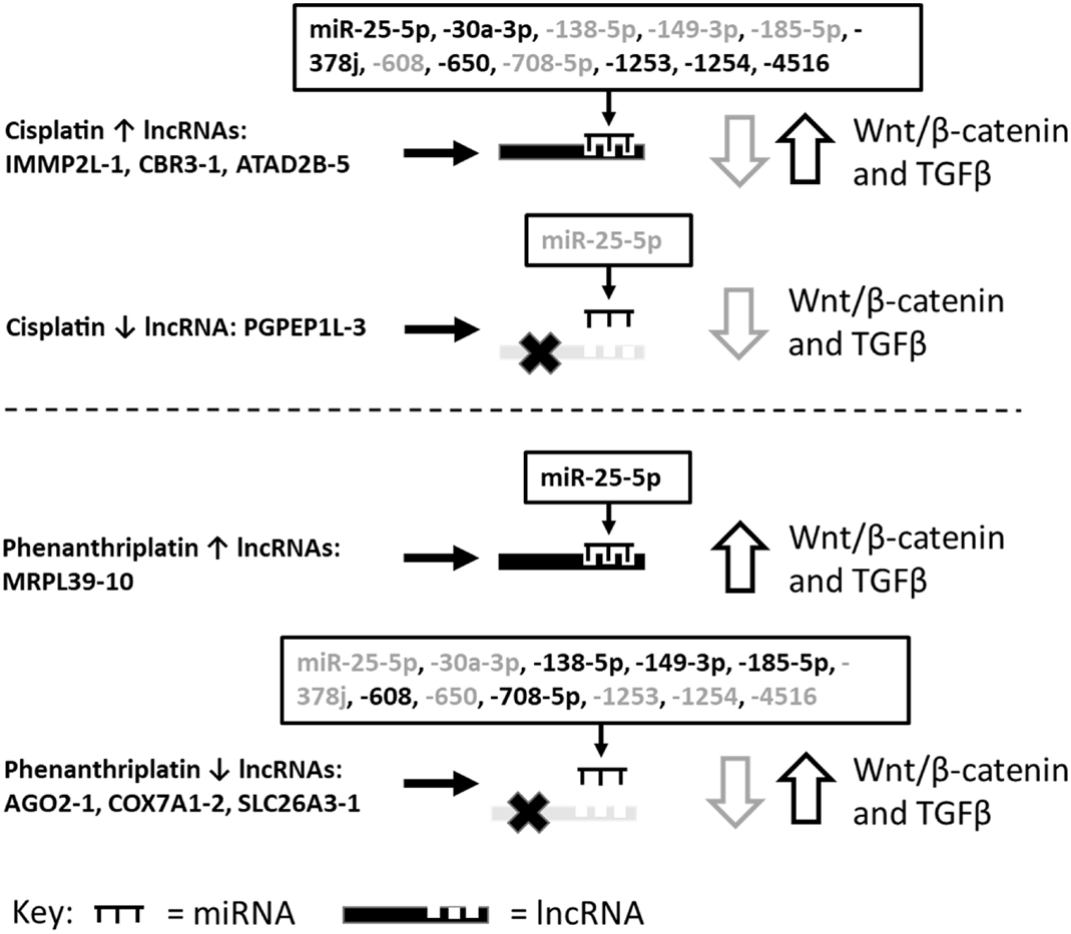
Potential modulation of Wnt/β-catenin and TGF-β signaling via cisplatin and phenanthriplatin regulation of lncRNAs and predicted miRNA binding partners in A549 cells. Key: Grey miR text = miRNA associated with downregulated Wnt/β-catenin and TGF-β signaling, Black miR text = miRNA associated with upregulated Wnt/β-catenin and TGF-β signaling, grey open down arrow = downregulated, black open up arrow = upregulated.

**Figure 5:**
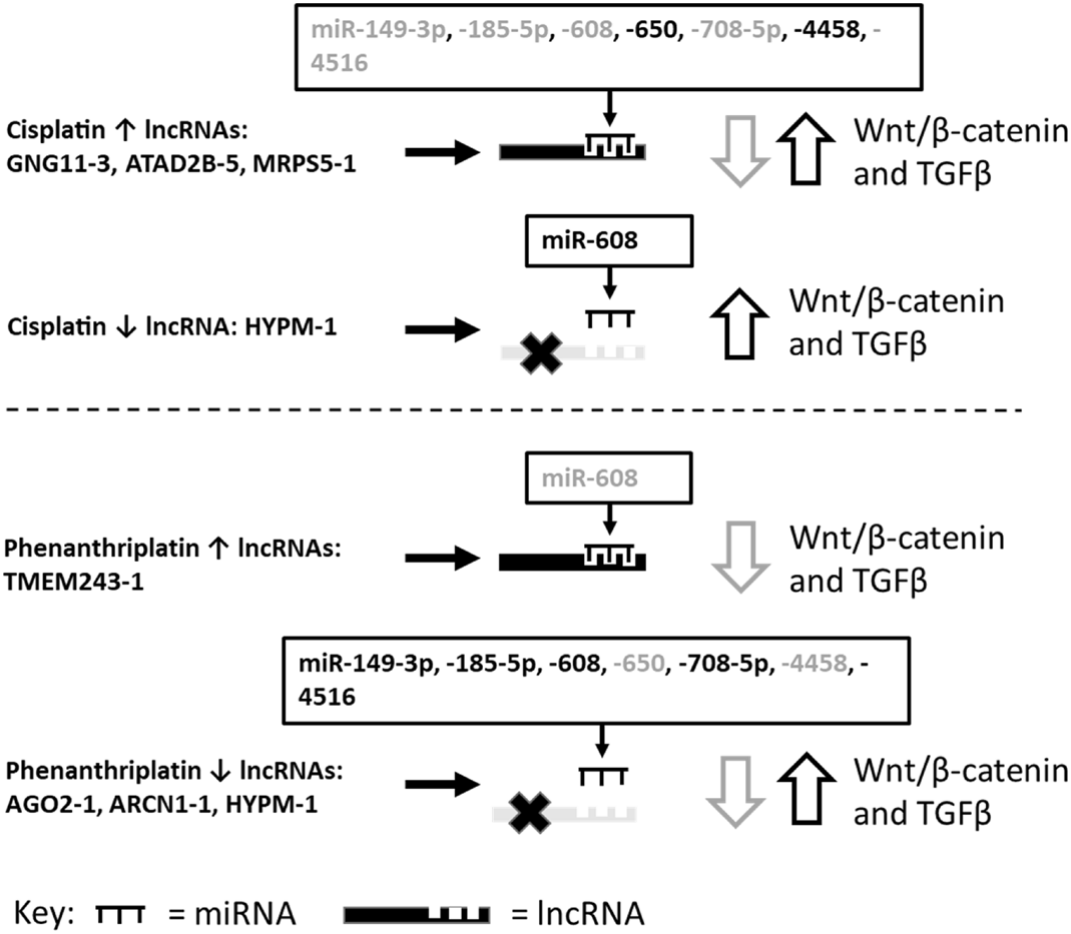
Potential modulation of Wnt/β-catenin and TGF-β signaling via cisplatin and phenanthriplatin regulation of lncRNAs and predicted miRNA binding partners in IMR90 cells. Key: Grey miR text = miRNA associated with downregulated Wnt/β-catenin and TGF-β signaling, Black miR text = miRNA associated with upregulated Wnt/β-catenin and TGF-β signaling, grey open down arrow = downregulated, black open up arrow = upregulated.

As a final step, we also analyzed how cisplatin and phenanthriplatin regulated lncRNAs might target miRNAs and potentially regulate Wnt/β-catenin and TGFβ signaling in both A549 (Fig. 4) and IMR90 cells (Fig. 5).

## Discussion

Cisplatin can act through lncRNAs that regulate disease progression in lung cancer by sponging miRNAs, whose interaction with lncRNAs modulates a variety of genes in cancer apoptosis, migration, proliferation and resistance^37–42^. As the role of lncRNAs in the anticancer effect of monofunctional platinum(II) complexes had not yet been assessed, we used microarray analysis to identify regulated lncRNAs in A549 and IMR90 cells after treatment with cisplatin or phenanthriplatin (Tables 1, 2). To validate our microarray results, we performed qRT-PCR on total RNA extracts from cisplatin and phenanthriplatin treated cell preparations and found that the relative fold change expression of regulated lncRNAs calculated via PCR amplification generally corroborated the microarray data (Fig. 2). Next, we applied bioinformatics methods to the microarray results and discovered that a set of miRNAs predicted to target the three most upregulated lncRNAs by cisplatin treatment also had high sequence homology to the three most downregulated lncRNAs, AGO2-1, COX7A1-2 and SLC26A3-1, after phenanthriplatin treatment (Tables 1, 3). Then, we used LNA qRT-PCR to comparatively measure the effect of cisplatin and phenanthriplatin treatment on the expression of several miRNAs that are predicted to be targeted by these lncRNAs (Fig. 3). These results suggest that cisplatin might upregulate a set of lncRNAs that sponge and nullify the gene regulatory effect of these miRNAs; whereas, phenanthriplatin, by reducing the expression of lncRNAs that could prevent the action of the same miRNAs, might promote miRNA gene silencing. The results also suggest that cisplatin and phenanthriplatin treatment might cause distinct regulation of miRNAs, particularly in A549 cells.

Several of the miRNAs detected in our analysis are associated with Wnt/β-catenin signaling. Expression of miR-25-5p may be targeted by two lncRNAs regulated by phenanthriplatin, e.g., lnc-COX7A1-2, that the monofunctional complex reduces, and lnc-MRPL39-10, which phenanthriplatin increases (Table 3). The sponging of miR-25-5p via increased lnc-MRPL39-10 expression could promote anticancer effect through the Wnt pathway as the reduction of this miRNA in A549 cells is associated with decreased cell viability, invasion and migration by decreasing Wnt signaling (Fig. 4)^25^. Our miRNA qRT-PCR results may corroborate lnc-MRPL39-10 suppression of miR-25-5p as phenanthriplatin treated A549 cells had decreased miR-25-5p expression compared to cisplatin treated samples (Fig. 3a).

As phenanthriplatin reduced the expression of the lncRNAs, AGO2-1, COX7A1-2, and SLC26A3-1 (Table 3), this could promote the action of miRNAs that might be sponged by these lncRNAs. Increased miR-149-3p and miR-185-5p expression downregulates Wnt/β-catenin signaling reducing cancer proliferation in A549 and lung adenocarcinoma cells^33,34^. Overexpression of miR-608 induced cell death in A549 cells via signaling that integrates the Wnt pathway^23^. MiR-708-5p expression also suppressed DNA methylation decreasing Wnt/β-catenin signaling and impairing the stemness characteristics of NSCLC cells^26^. Therefore, phenanthriplatin could act to suppress NSCLC by repressing lncRNAs that regulate miR-25-5p, -185-5p, -608, and -708-5p, through the Wnt/β-catenin pathway (Fig. 4).

However, increasing the expression of some of the miRNAs may also promote cancer through the Wnt/β-catenin pathway. Suppression of miR-378 in A549 cells inactivates Wnt/β-catenin signaling leading to reduced cell proliferation^28^, and as phenanthriplatin treatment caused reduced miR-378j compared to cisplatin (Fig. 3a), the monofunctional complex may act through this pathway to prevent cancer proliferation. MiR-650 can be upregulated in A549 cells where it promoted proliferation and invasion through the Wnt-1/β‑catenin pathway by acting on inhibitor of growth 4^29^. Increased expression of miR-1253, which directly targets Wnt5A, is associated with the proliferation, migration, and invasion of NSCLC cells^27^. Similarly, miR-1254 targets the Wnt/β-catenin pathway antagonist, secreted frizzled related protein 1 (SFRP1), and was upregulated in lung cancer tissues and cells promoting proliferation^24^. Our results suggest that the monofunctional complex might act against NSCLC via Wnt/β-catenin signaling if the miRNAs, miR-149-3p, -185-5p, -608, and -708-5p were not sponged by the phenanthriplatin downregulated lncRNAs, AGO2-1, COX7A1-2 and SLC26A3-1. Nonetheless, increased expression of the miRs, -25-5p, -650, -1253, and -1254, when these lncRNAs are suppressed by the monofunctional complex, could promote cancer by modulating the Wnt/β-catenin pathway possibly in conjunction with other pathway signaling (Fig. 4).

LncRNAs are also able to act in NSCLC through the TGF-β pathway and integrated MAPK/ERK and PI3K/AKT signaling^43,44^. We identified several miRNAs that might integrate TGF-β signaling components through lncRNAs regulated by phenanthriplatin (Fig. 4). Overexpression of miR-608 induced cell death in A549 cells through the MAPK/ERK pathway^23^. As the three lncRNAs, AGO2-1, COX7A1-2, and SLC26A3-1, which are predicted to sponge miR-608, were downregulated by the monofunctional complex (Table 3), we would expect that expression of this miRNA would lead to NSCLC cell death via ERK signaling. However, suppression of AGO2-1, COX7A1-2, and SLC26A3-1, could also have an opposite effect in NSCLC cells by upregulating the miRs, -25-5p, -30a-3p and -378, which act via MAPK/ERK, and are projected targets of these lncRNAs (Table 3), and have reduced anti-cancer efficacy when their expression is increased^25,28,30^. We found that phenanthriplatin treatment downregulated miR-378j more than cisplatin (Fig. 3a), which suggests that the monofunctional complex may signal via the TGF-β/MAPK/ERK pathway to promote anti-cancer effect. As miR-25-5p may be sponged via phenanthriplatin-mediated upregulation of lnc-MRPL39-10 (Table 3), this miRNA could be downregulated in A549 cells, which is associated with anti-cancer effect via PI3K signaling^25^. Reduction of AGO2-1, COX7A1-2, and SLC26A3-1 by phenanthriplatin would also be expected to lead to increased miR-138-5p and -608, whose expression modulates PTEN/PI3K/AKT signaling and is associated in A549 cells with anticancer effect^23,31,45–47^. In retinoblastoma tumors, increased miR-4516 expression promotes tumor growth through the PTEN/AKT pathway^32^. As phenanthriplatin treatment caused increased miR-4516 expression in A549 cells compared to cisplatin (Fig. 3a), the monofunctional complex may be able to promote PTEN/AKT signaling more than its bifunctional counterpart.

We also examined whether cisplatin and phenanthriplatin might regulate Wnt/β-catenin and TGF-β signaling differently in IMR90 compared to A549 cells through lncRNA regulation of miRNAs. Interestingly, a majority of the miRNAs, e.g., miR-149-3p, -185-5p, -608, -650, -708-5p, and -4516, identified as potential targets of lncRNAs in A549 cells, were predicted to be regulated identically in IMR90 cells albeit by different lncRNAs (Table 4, Fig. 5). Specifically, cisplatin upregulates lncRNAs, e.g., ATAD2B-5, GNG11-3, and MRPS5-1, that collectively should sponge all of these miRNAs, and phenanthriplatin downregulates lncRNAs, e.g., AGO2-1, ARCN1-1, and HYPM-1, that should allow expression of these miRNAs. Cisplatin also downregulates HYPM-1, which should allow expression of miR-608; whereas, the monofunctional complex upregulates TMEM243-1, a lncRNA with high sequence homology to this miRNA. Therefore, regulation of Wnt/β-catenin and TGF-β signaling through miR-149-3p, -185-5p, -608, -650, -708-5p, and -4516 would be predicted to be similar in both IMR90 and A549 cells. However, it is possible that simultaneous upregulation of multiple lncRNAs, e.g., ATAD2B-5 and MRPS5-1 in cisplatin treated IMR90 cells, and downregulation of multiple lncRNAs, e.g., AGO2-1, ARCN1-1, and HYPM-1 by phenanthriplatin, could synergistically promote or prevent respectively the action of other lncRNAs and might alter Wnt/β-catenin and TGF-β signaling differently than would occur from the effect of these lncRNAs and miRNAs alone. When we measured miR-149-3p and -608 expression in the IMR90 cell line, we found that cisplatin and phenanthriplatin did not exhibit distinct regulation of either miRNA (Fig. 3b). Thus, it is possible that the action of specific lncRNAs to modulate miRNA expression, and Wnt/β-catenin and TGF-β signaling, could be masked by the effect of larger combinations of up- and downregulated lncRNAs acting together.

Further, we also identified a potential miRNA target, miR-4458, unique to IMR90 cells. Cisplatin treatment upregulated lncRNA, ATAD2B-5, which might sponge miR-4458, while phenanthriplatin downregulated both AGO2-1 and HYPM-1, lncRNAs with targeting homology to this miRNA (Table 4). Interestingly, cisplatin treatment in IMR90 cells caused increased miR-4458 expression compared to phenanthriplatin (Fig. 3b), which could mean that increased ATAD2B-5 is less effective as a suppressor of this miRNA than the effect of decreasing either AGO2-1 or HYPM-1. As increased expression of miR-4458 prevents proliferation and migration in NSCLC cell lines via the PI3K/AKT pathway^36^, cisplatin treatment might suppress miR-4458 with ATAD2B-5 allowing cancer progression, while phenanthriplatin, by decreasing the expression of AGO2-1 and HYPM-1, could allow miR-4458 expression through modulating the PI3K/AKT pathway to promote anti-cancer activity.

As in A549 cells, phenanthriplatin regulated a distinct set of lncRNAs compared to cisplatin in IMR90 cells (Tables 1, 2) and might modulate Wnt/β-catenin and TGF-β signaling differently in normal lung fibroblast cells than in lung cancer cells (Figs. 4, 5). In A549 cells, miR-25-5p expression may be sponged by upregulated lnc-MRPL39-10, but we found in IMR90 cells that this miRNA would be potentially decoyed by lnc-HYPM-1, which is downregulated by phenanthriplatin (Tables 2, 3, 4). The absence of suppression of miR-25-5p by lnc-HYPM-1 in IMR90 cells could promote cell proliferation and viability via Wnt/β-catenin and/or TGF-β signaling integrating MAPK/ERK as found in A549 cells^25^. Similarly, miR-138-5p and miR-608 are potentially not subject to sponging in A549 cells treated with the monofunctional complex as their lncRNA targets are downregulated, but in IMR90 cells, these miRNA could be sponged by upregulated lnc-TMEM243-1 (Tables 2, 3, 4). Therefore, reduced expression of miR-138-5p and -608 due to TMEM243-1 decoying in IMR90 cells could lead to reduced cytotoxicity via TGF-β signaling (miR-138-5p) or Wnt/β-catenin and TGF-β signaling (miR-608) as suggested by the effects of suppressing these miRNAs in A549 cells^23,46–49^.

## Materials and methods

### Cell culture and treatment

The A549 and IMR90 cell lines were obtained from ATCC (Manassas, VA). A549 cells were cultured in F12K media with 10% FBS and 1% penicillin/streptomycin supplementation. IMR90 cells were cultured in Eagle's Minimum Essential Medium (EMEM) with 10% FBS and 1% penicillin/streptomycin supplementation. We seeded 6-well dishes with 3 × 10^5^ cells per well and placed them in an incubator (37 °C, 5% CO_2_) for 24 h. Sets of three wells each were treated with either a negative control (media only), 5 µM cisplatin (Sigma-Aldrich, St. Louis, MO), or 5 µM phenanthriplatin (synthesized by Dr. Kevin M. Williams, Western Kentucky University Department of Chemistry, Bowling Green, KY according to a published protocol^8^), a platinum concentration that efficiently targets the nucleus at 24 h^8^. Then, the dishes were placed back into the incubator for 24 h after which they were prepared for RNA isolation.

### RNA isolation

The Qiagen RNeasy kit (Hilden, Germany) was used to isolate RNA samples from control and platinum complex-treated dishes per kit instructions. Media was aspirated out of the wells followed by washing (1×) with phosphate buffered saline (PBS). The PBS was removed, and Buffer RLT Plus with β-mercaptoethanol was introduced into each well and homogenate was then transferred to a microcentrifuge tube. Each tube was vortexed and the lysate was transferred to a Gdna Eliminator spin column and centrifuged. Flow-through was collected and 70% ethanol was added. The samples were then transferred to an RNeasy spin column placed in a collection tube. Spin columns were centrifuged and the flow-through was discarded. Buffer RW1 was added to each spin column, followed by centrifugation. Flow-through was discarded and Buffer RPE with ethanol was added followed by centrifugation. The flow-through was again discarded and Buffer RPE with ethanol was then added followed by centrifugation. The spin column was then placed in a collection tube, centrifuged, and then placed in a new collection tube. Two elutions using RNase-free water were performed with centrifugation. All RNA samples were stored at − 80 °C for subsequent analysis.

### Microarray assay

RNA transcription profiles from experimental and control samples were created using a microarray assay. RNA samples were analyzed using a NanoDrop 2000 (Thermo Fisher, Waltham, MA) and quality was assessed with an Agilent 2100 Bioanalyzer (Agilent, Wilmington, DE). RNA integrity was expressed as RNA integrity number (RIN) values and only samples with values greater than 10 were used. The Low RNA Input Linear Amplification kit with one-color (Agilent, Wilmington, DE) was used to produce fluorescent cRNA. Labeled cRNA was then purified using the RNeasy Mini Elute kit (Qiagen, Valencia, CA). Fluorescent cRNA samples were then hybridized onto Whole Human Genome 4 × 44 K microarrays GeneChips (Affymetrix, Santa Clara, CA). Four replicates were performed with array hybridization conducted for 17 h at 65 °C. Slides were then scanned with an Agilent microarray scanner (G2565BA) using Feature Extraction software (v 9.5.1, Agilent). GeneSpring GX 10.0.2 software (Agilent Technologies) was used for statistical analysis, background correction, normalization and summary of expression measure.

### Quantitative real-time and locked nucleic acid polymerase chain reaction analysis

Microarray results were additionally validated using quantitative real time-polymerase chain reaction (qRT-PCR) on RNA samples also used for the microarrays. Custom probes were designed and specificity checked using the National Institutes of Health Primer-BLAST tool and obtained from Integrated DNA Technologies (Coralville, IA). Samples were placed in an Agilent Technologies Stratagene Mx3000P real-time PCR System (Santa Clara, CA) with MxPro software with thermal cycling at 95 °C for 10 min, followed by 40 cycles of denaturing at 95 °C for 30 s, annealing at 55 °C for 30 s, and extension at 72 °C for 30 s. Additionally, RNA samples were analyzed using locked nucleic acid PCR (LNA) miRNA to measure the expression of miRNA targets (A549: miR-25-5p, -378j, -4516; IMR90: miR-149-3p, -608, -4458). Custom LNA probes were obtained from Qiagen (Hilden, Germany). Samples were analyzed with the Mx3000P and MxPro software as per the qRT-PCR experiments except that thermal cycling was performed at 95 °C for 2 min (initial activation), followed by 40 cycles of denaturing at 95 °C for 10 s, and annealing/extension at 56 °C for 1 min. Three independent reactions were performed for each qRT-PCR and LNA PCR experiment with three technical replicates prepared in each. Quantitative data for qRT-PCR and LNA PCR experiments were normalized to the rRNA 5S gene.

### Data and statistical analysis

Long noncoding RNA transcript IDs identified by the microarray assay were sequence characterized using the LNCipedia database (https://lncipedia.org/^50^. Identification of miRNA targeting of lncRNAs used the RNA22 v2 microRNA target detection software (https://cm.jefferson.edu/rna22/Interactive/^51^ with *p* ≤ 0.05 as the standard (except that for GNG11-3, *p* ≤ 0.0581). Microarray data was statistically analyzed using GeneSpring GX 10.0.2 software (Agilent Technologies) with *p* ≤ 0.05 as the standard. qRT-PCR and LNA PCR data were analyzed using the Livak equation to determine relative fold values and standard deviation fold change was calculated using the formula: s = ((s_1_)^2^ + (s_2_)^2^)^1/2^. GraphPad PRISM (version 9.0.0; La Jolla, CA) was used for all other statistical analysis with *p* ≤ 0.05 as the standard.

## Supplementary Information

Supplementary Tables.

## Data Availability

The datasets generated during and/or analyzed during the current study are available from the corresponding author on reasonable request.
