# Supplementary material for: Cisplatin and phenanthriplatin modulate long-noncoding RNA expression in A549 and IMR90 cells revealing regulation of microRNAs, Wnt/β-catenin and TGF-β signaling
Source: Sci Rep. 2021 May 17;11:10408. doi: 10.1038/s41598-021-89911-z (PMC8129125; doi:10.1038/s41598-021-89911-z)
Supplement: Supplementary file 1 — Supplementary Tables. [file 41598_2021_89911_MOESM1_ESM.pdf]

Title: Cisplatin and phenanthriplatin modulate long-noncoding RNA expression in A549 and IMR90 cells revealing regulation of microRNAs, Wnt/β-catenin and TGF-β signaling

Authors: Jerry D. Monroe, Satya A. Moolani, Elvin N. Irihamye, Katheryn E. Lett, Michael D. Hebert, Yann Gibert, Michael E. Smith

Supplementary Table 1: Regulated lncRNAs and microRNA targets in the A549 cell line			
A549 cisplatin vs. A549 control-Upregulated			
lnc-IMMP2L-1			
Identifier	Predicting Target Site	Targeting mRNA	p value
hsa_miR_1253	AACAATACAATCTTCTTGCTCT	AGAGAAGAAGATCAGCCTGCA	0.00333
hsa_miR_1254	TCTTCTTGCTCTGTCACCCAGGCT	AGCCTGGAAGCTGGAGCCTGCAGT	0.00333
hsa_miR_30a_3p	CTTGCTCTGTCACCCAGGCTGAAAT	CTTTCAGTCGGATGTTTGCAGC	0.00333
hsa_miR_650	ATCTTCTTGCTCTGTCACCC	AGGAGGCAGCGCTCTCAGGAC	0.00333
lnc-CBR3-1			
Identifier	Predicting Target Site	Targeting mRNA	p value
hsa_miR_25_5p	CAGTTTGCAGGGAGCTCAGCTC	AGGCGGAGACTTGGGCAATTG	0.00058
hsa_miR_378j	AAAAGGCCAAGAATTCAGT	ACTGGATTTGGAGCCAGAA	0.00058
hsa_miR_650	CCCCTTTAGAGTTGTAAGCCTTTA	AGGAGGCAGCGCTCTCAGGAC	0.00058
hsa_miR_650	GTTTGCAGGGAGCTCAGCTCTT	AGGAGGCAGCGCTCTCAGGAC	0.00058
hsa_miR_708_5p	CCCTTTAGAGTTGTAAGCCTTT	AAGGAGCTTACAATCTAGCTGGG	0.00058
lnc-ATAD2B-5			
Identifier	Predicting Target Site	Targeting mRNA	p value
hsa_miR_149_3p	GCCACCCACTGGCTTCTCT	AGGGAGGGACGGGGGCTGTGC	0.00675
hsa_miR_185_5p	CCACCCACTGGCTTCTCTCACG	TGGAGAGAAAGGCAGTTCCTGA	0.00675
hsa_miR_4516	GCCACCCACTGGCTTCTCTC	GGGAGAAGGGTCGGGGC	0.00675
hsa_miR_608	CACTGGCTTCTCTCACGGCCACCCTG	AGGGGTGGTGTTGGGACAGCTCCGT	0.00675
A549 phenanthriplatin vs. A549 control-Upregulated			
lnc-MRPL39-10			
Identifier	Predicting Target Site	Targeting mRNA	p value
hsa_miR_25_5p	TAATATACAAGTTTCCACTG	AGGCGGAGACTTGGGCAATTG	0.019
A549 cisplatin vs. A549 control-Downregulated			
lnc-PGPEP1L-3			
Identifier	Predicting Target Site	Targeting mRNA	p value
hsa_miR_25_5p	TGTATGCCCACACCCTCTTCTC	AGGCGGAGACTTGGGCAATTG	7.7E-05

<u>**A549 phenanthriplatin vs. A549 control-Downregulated**</u>			
**lnc-AGO2-1**			
Identifier	Predicting Target Site	Targeting mRNA	p value
hsa_miR_149_3p	CTCTCTCCCCCTCTCCCCCCT	AGGGAGGGACGGGGGCTGTGC	1E-06
hsa_miR_149_3p	CCTCTCCCCCCTCCCCCTCCCT	AGGGAGGGACGGGGGCTGTGC	1E-06
hsa_miR_185_5p	TCTCTCTCTCTCTCTCTCTCCC	TGGAGAGAAAGGCAGTTCCTGA	1E-06
hsa_miR_4516	CTCTCTCCCCCTCTCCC	GGGAGAAGGGTCGGGGC	1E-06
hsa_miR_608	CTCTCTCTCTCCCCCTCTCCCCCCT	AGGGGTGGTGTTGGGACAGCTCCGT	1E-06
**lnc-SLC26A3-1**			
Identifier	Predicting Target Site	Targeting mRNA	p value
hsa_miR_1253	TGCGGCGCAGCTGCCTCCTCT	AGAGAAGAAGATCAGCCTGCA	0.0465
hsa_miR_138_5p	TGGAGGGTGGAGAGCCAGTG	AGCTGGTGTTGTGAATCAGGCCG	0.0465
hsa_miR_149_3p	GCGCAGCTGCCTCCTCTTA	AGGGAGGGACGGGGGCTGTGC	0.0465
hsa_miR_30a_3p	GCGCCAGGGCCGGATGGAGG	CTTTCAGTCGGATGTTTGCAGC	0.0465
hsa_miR_378j	ACTGGGCGCCAGGGCCGGA	ACTGGATTTGGAGCCAGAA	0.0465
hsa_miR_608	CCAGTGCGGCGCAGCTGCCTCCTCT	AGGGGTGGTGTTGGGACAGCTCCGT	0.0465
hsa_miR_650	CAGTGCGGCGCAGCTGCCTCCT	AGGAGGCAGCGCTCTCAGGAC	0.0465
hsa_miR_708_5p	GCCAGTGCGGCGCAGCTGCCTC	AAGGAGCTTACAATCTAGCTGGG	0.0465
**lnc-COX7A1-2**			
Identifier	Predicting Target Site	Targeting mRNA	p value
hsa_miR_1254	ACTGCAAAGCTCCGCCTCCCAGGTT	AGCCTGGAAGCTGGAGCCTGCAGT	0.0487
hsa_miR_25_5p	TCACTGCAAAGCTCCGCCT	AGGCGGAGACTTGGGCAATTG	0.0487
hsa_miR_608	TCGGCTCACTGCAAAGCTCCGCCTCC	AGGGGTGGTGTTGGGACAGCTCCGT	0.0487
hsa_miR_650	CTCACTGCAAAGCTCCGCCTCCC	AGGAGGCAGCGCTCTCAGGAC	0.0487

**Supplementary Table 2: Regulated lncRNAs and microRNA targets in the IMR90 cell line**			
<u>**IMR90 cisplatin vs. IMR90 control-Upregulated**</u>			
**lnc-GNG11-3**			
Identifier	Predicting Target Site	Targeting mRNA	p value
hsa_miR_149_3p	ACATGAGATTTCAGCTCTCCTG	AGGGAGGGACGGGGGCTGTGC	0.0581
hsa_miR_650	AACATGAGATTTCAGCTCTCCT	AGGAGGCAGCGCTCTCAGGAC	0.0581
**lnc-ATAD2B-5**			
Identifier	Predicting Target Site	Targeting mRNA	p value
hsa_miR_185_5p	CCACCCACTGGCTTCTCTCACG	TGGAGAGAAAGGCAGTTCCTGA	0.0068

hsa_miR_4458	AGCCACCCACTGGCTTCT	AGAGGTAGGTGTGGAAGAA	0.0068
hsa_miR_4516	GCCACCCACTGGCTTCTCTC	GGGAGAAGGGTCGGGGC	0.0068
hsa_miR_608	CACTGGCTTCTCTCACGGCCACCCTG	AGGGGTGGTGTTGGGACAGCTCCGT	0.0068
hsa_miR_708_5p	CCCAAAGCCACCCACTGGCTTCTC	AAGGAGCTTACAATCTAGCTGGG	0.0068
**lnc-MRPS5-1**			
Identifier	Predicting Target Site	Targeting mRNA	p value
hsa_miR_608	GAACAAACAACCCACATCTACACCCA	AGGGGTGGTGTTGGGACAGCTCCGT	0.0151
**IMR90 phenanthriplatin vs. IMR90 control-Upregulated**			
**lnc-TMEM243-1**			
Identifier	Predicting Target Site	Targeting mRNA	p value
hsa_miR_608	CCGGTTGCAGTTCACAAAATACACTTTG	AGGGGTGGTGTTGGGACAGCTCCGT	0.0009
**IMR90 cisplatin vs. IMR90 control-Downregulated**			
**lnc-HYPM-1**			
Identifier	Predicting Target Site	Targeting mRNA	p value
hsa_miR_608	TTGGAACAACCCTCTCCAGCCCA	AGGGGTGGTGTTGGGACAGCTCCGT	0.0162
**IMR90 phenanthriplatin vs. IMR90 control-Downregulated**			
**lnc-AGO2-1**			
Identifier	Predicting Target Site	Targeting mRNA	p value
hsa_miR_149_3p	CTCTCTCCCCCTCTCCCCCCT	AGGGAGGGACGGGGGCTGTGC	1E-06
hsa_miR_149_3p	CCTCTCCCCCCTCCCCCTCCCT	AGGGAGGGACGGGGGCTGTGC	1E-06
hsa_miR_185_5p	TCTCTCTCTCTCTCTCTCTCCC	TGGAGAGAAAGGCAGTTCCTGA	1E-06
hsa_miR_4458	CCCCTCCCCCTCCCTCT	AGAGGTAGGTGTGGAAGAA	1E-06
hsa_miR_4516	CTCTCTCCCCCTCTCCC	GGGAGAAGGGTCGGGGC	1E-06
hsa_miR_608	CTCTCTCTCTCCCCCTCTCCCCCCT	AGGGGTGGTGTTGGGACAGCTCCGT	1E-06
**lnc-HYPM-1**			
Identifier	Predicting Target Site	Targeting mRNA	p value
hsa_miR_149_3p	GTATTTCCCTCCTCTCCCC	AGGGAGGGACGGGGGCTGTGC	0.0103
hsa_miR_4458	CTCTCCCCCATTTTCCTCC	AGAGGTAGGTGTGGAAGAA	0.0103
hsa_miR_4516	TTCCCTCCTCTCCC	GGGAGAAGGGTCGGGGC	0.0103
hsa_miR_608	TTGGAACAACCCTCTCCAGCCCA	AGGGGTGGTGTTGGGACAGCTCCGT	0.0162
hsa_miR_708_5p	TCCAGCCCAGGACATGCCAGCGCTTA	AAGGAGCTTACAATCTAGCTGGG	0.0162

hsa_miR_185_5p	CCTCCCTCTGGCTCTCTCCA	TGGAGAGAAAGGCAGTTCCTGA	0.0362
hsa_miR_650	AGCCTGTGTCTGTCTGCCTTTG	AGGAGGCAGCGCTCTCAGGAC	0.0362
hsa_miR_650	GTCTGCCTTTGCTTCCTCCC	AGGAGGCAGCGCTCTCAGGAC	0.0362
**lnc-ARCN1-1**			
Identifier	Predicting Target Site	Targeting mRNA	p value
hsa_miR_149_3p	GCAACCTCCACCTCCCA	AGGGAGGGACGGGGGCTGTGC	0.001
hsa_miR_185_5p	TCACCTCACTGCAACCTCCA	TGGAGAGAAAGGCAGTTCCTGA	0.001
hsa_miR_608	GATTCCTCTTCCCACACCCACCCTG	AGGGGTGGTGTTGGGACAGCTCCGT	0.0016
